# Proceedings of the Buffalo Medical Association

**Published:** 1877-11

**Authors:** E. N. Brush

**Affiliations:** Sec’y.


ART. III.—*Abstract of the Proceedings of the Buffalo Medical Association,* Tuesday, Oct. 3d, 1877. Reported by E. N. Brush, M. D., Sec'y.

The Vice-President, Dr. Lothrop occupied the chair.

The minutes of the previous meeting were read and approved.

Under the head of Reports from Special Committees Dr. Howe presented a schedule of papers to be read before the Association for the ensuing seven months together with some recommendations as to the mode of conducting the discussion of the papers read. The report was received and upon motion adopted.

Dr. Mynter the essayist for the evening read a paper upon the "Mechanical Treatment of certain forms of Pulmonary Disease." Dr. Mynter in his paper referred to the use of rarified and compressed air in the treatment of Pulmonary emphysema, asthma, phthisis, etc., and illustrated his remarks by exhibiting the apparatus employed and explaining its uses.

At the conclusion of the paper the Chairman called upon the various members to discuss the points brought forth.

Dr. Rochester said that the subject was one with which he was not very familiar either in a practical or theoretical way. He had not seen very much of the literature of the subject, and while he was aware of its being employed as Dr. Mynter had stated in his paper, he had never taken pains to investigate its value. He did not, however, see why emphysema could not be treated in this manner and should look upon it in certain cases as a valuable agent. The Doctor had in his paper reported some successful applications of the apparatus, he did not desire to question his diagnosis but as most of the gentlemen present were aware there had been during the late summer and early fall months considerable hay asthma which in some of its forms might be mistaken by an enthusiastic observer for emphysema. The reader was a young man who, like the majority of young practioners might be apt to become enthusiastic over a theory and see in every case of lung trouble a case to be treated by his method. The apparatus doubtless had value and Dr. Mynter deserved credit for investigating and experimenting with it, and if over sanguine as to its merits would have ample time to revise his opinions.

Dr. Strong said the apparatus struck him as a means of treatment which might be of value. Thought it might be useful in the treatment of incipient tuberculosis. It had often occurred to him that certain forms of lung disease should be met more directly than was the usual custom, and the method thus advanced of treating emphysema seemed an advance in the right direction.

Dr. Bartlett remarked that the instrument was to him a novelty. He thought that back of all lung diseases as manifested by their symptoms there were certain anatomical changes which it was necessary to take into consideration in using either rarified or compressed air and that they should be taken into careful consideration lest harm be done by injudicious interference.

Dr. J. F. Miner said he had been much interested in the paper or that portion of it which he had been able to understand. Dr. Mynter had presented an apparatus which he had seen represented substantially as presented, for years. He understood the doctor to say the machine would register the lung capacity for air, and to intimate that our young friend, Dr. Cary, had more wind than the average of doctors, which, without trial, he thought hardly fair. An instrument to measure our wind might be a good thing. To be serious he had not given the matter any attention, looking upon it as something over which one could easily grow sanguine of success but by age and more extended experience become convinced that their earlier views needed modification.

Dr. White said he would content himself with saying that the Association should be under obligations to Dr. Mynter who had at no small expense of time and trouble been able to exhibit the apparatus. It was evident, that few present had any practical knowledge of the subject, although it was one not entirely novel to him he knew comparatively little about it. It does not matter whether all the hopes which the author expresses in his paper are accomplished or not, it is important to the members of the Association that they do not remain ignorant of the subject or of the results which it seeks and claims to accomplish.

Dr. W. W. Miner said that a similar instrument was used at Amherst College for testing the lungs of students in the classes at the gymnasium and record was made annually of the expulsive force of the chest, the inspiratory power of the lungs and the volume of air that could be expelled after the fullest inspiration. The instruments were open for use of the students throughout the year and it was found that exercise would considerably increase the expulsive power of the lungs but would not affect the vital capacity or expansive power in the same ratio.

Dr. Hopkins expressed some surprise that the majority of those present had called this a novelty. It was on the contrary something which had been for some time known and described. He did not wish to enter into a discussion of the therapeutic value of the method but desired to congratulate Dr. Mynter. He would congratulate the Doctor first upon the commercial value of the apparatus. It had its moral force and enabled him to make an impression upon the pockets of the patients as well as upon their ailments. He would congratulate him also upon the therapeutic value of the instrument as one of the successful cases reported in his paper had applied to him and had been treated but without benefit. The Doctor was also to be congratulated upon his boldness in taking up a matter of this kind and thus preserving for the regular profession something which might easily be usurped by the quacks.

Dr. Van Peyma said that he knew nothing of the subject but from what he had heard during the evening was favorably impressed therewith.

The discussion was closed by some explanatory remarks by Dr. Mynter.

Dr. White said that he had a specimen which he desired to present, it consisted of the neck of the uterus which he had amputated for cancerous diseases the day previous. His views upon the treatment of cancer involving the os and neck of the uterus had undergone some change within the last few years, and he now thought that these affections demanded energetic treatment. The specimen in question was removed from a German woman sent to him by Dr. Hauenstein and was amputated by the assistance of Dr. Brush with the galvano-cautery. (The report of this case will be presented in detail in a subsequent number of the *Journal.*)

The introduction of this case gave rise to some interesting discussions upon the advisability of operating upon cancerous affections but the discussion was of such desultory character that the secretary was unable to report it in such manner as to do justice to the speakers.

Dr. W. W. Miner exhibited under the microscope a preparation from cancer of the uterus which gave rise to a discussion upon "cancer cells."

The Secretary was instructed to have printed lists of the papers to be read at the ensuing meetings prepared for distribution.

On motion the meeting adjourned.

